# Exploring oak processionary caterpillar induced lepidopterism (part 2): ex vivo bio-assays unmask the role of TRPV1

**DOI:** 10.1007/s00018-024-05318-9

**Published:** 2024-06-28

**Authors:** Andrea Seldeslachts, Eivind Andreas Baste Undheim, Joris Vriens, Jan Tytgat, Steve Peigneur

**Affiliations:** 1 Toxicology and Pharmacology, Department Pharmaceutical and Pharmacological Sciences, KU Leuven, Leuven, Vlaams-Brabant, Belgium; 2 Centre for Biochemistry and Molecular Biology, Department of Biosciences, The University of Oslo, Oslo, Norway; 3 Laboratory of Endometrium, Endometriosis and Reproductive Medicine, Department of Development and Regeneration, KU Leuven, Leuven, Vlaams-Brabant, Belgium

**Keywords:** *Thaumetopoea processionea*, Contact dermatitis, Itch, TRPV1, Secapin-like peptide toxin and electrophysiology

## Abstract

As human skin comes into contact with the tiny hairs or setae of the oak processionary caterpillar, *Thaumetopoea processionea*, a silent yet intense chemical confrontation occurs. The result is a mix of issues: skin rashes and an intense itching that typically lasts days and weeks after the contact. This discomfort poses a significant health threat not only to humans but also to animals. In Western Europe, the alarming increase in outbreaks extends beyond areas near infested trees due to the dispersion of the setae. Predictions indicate a sustained rise in outbreaks, fueled by global changes favoring the caterpillar’s survival and distribution. Currently, the absence of an efficient treatment persists due to significant gaps in our comprehension of the pathophysiology associated with this envenomation. Here, we explored the interaction between the venom extract derived from the setae of *T. processionea* and voltage- and ligand-gated ion channels and receptors. By conducting electrophysiological analyses, we discovered ex vivo evidence highlighting the significant role of TPTX_1_-Tp1, a peptide toxin from *T. processionea*, in modulating TRPV1. TPTX_1_-Tp1 is a secapin-like peptide and demonstrates a unique ability to modulate TRPV1 channels in the presence of capsaicin, leading to cell depolarization, itch and inflammatory responses. This discovery opens new avenues for developing a topical medication, suggesting the incorporation of a TRPV1 blocker as a potential solution for the local effects caused by *T. processionea*.

**Supplementary Information:**

The online version contains supplementary material available at 10.1007/s00018-024-05318-9.

## Introduction

**Fig. 1:**
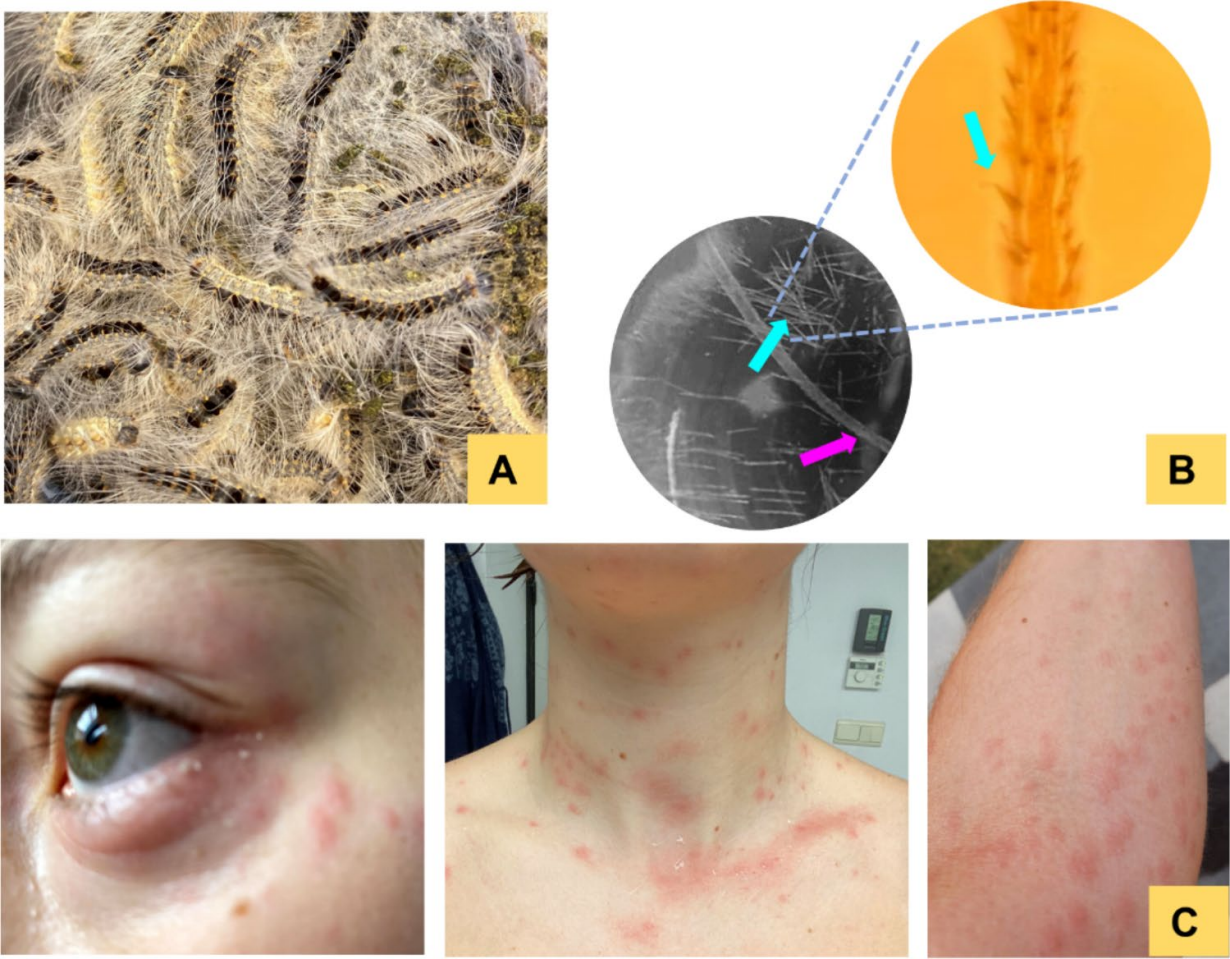
Lepidopterism caused by defensive setae of *Thaumetopoea processionea*. **A** Mature *T. processionea* larva covered with setae on an oak tree in Belgium. **B** Microscopic picture of stinging harpoon-shaped venomous setae (blue arrows) and the normal long smooth insect hairs (pink arrow). **C** Clinical picture: swelling and redness at conjunctive and urticaria on the skin of one of the authors following exposure to setae

In recent years, the processionary caterpillars have emerged as a significant ecological and public health concern in Europe [1]. Western Europe is home to three members of the ‘processionary’ species: *Thaumetopoea processionea* (oak processionary caterpillar), *Thaumetopoea pityocampa* (pine processionary caterpillar), and *Thaumetopoea pinivora* (northern pine processionary caterpillar) [2, 3]. Of particular interest is *T. processionea*, native to Belgium, The Netherlands, France, Germany, and the UK (Fig. 1A). The delicate yet purposeful fuzz covering *T. processionea* reveals a narrative of two distinct types of hairs—one ordinary, the other extraordinary [4]. While conventional smooth insect hairs provide protection, aid in environmental sensing, and contribute to the insect's overall structure, their furry harpoon-shaped venomous counterparts, also called setae, harbor bioactive molecules that tell a story of adaptation and survival (Fig. 1B) [4]. This survival mechanism incorporates a distinctive defense strategy wherein the caterpillar releases its setae into the environment upon sensing a threat [5]. The wind can blow these setae over a considerable distance where they remain active for approximately 10 years [5, 6]. Activities such as walking and biking near infested oak trees or swimming in water contaminated with the caterpillar’s urticating setae are sufficient for potential exposure. The consequences are far-reaching, with individuals facing lepidopterism—a form of irritant contact dermatitis characterized by the onset of an irresistible itch without lesions, followed by swelling, erythema, wheal, and flare reactions manifesting 6–8 h after the contact (Fig. 1C) [7]. Blister formation and ocular complications may occur occasionally, while inhalation may cause pharyngeal discomfort and respiratory distress [8–10]. The delayed onset of the reactions contributes to the complexity of the clinical profile. In severe cases, individuals may even face life-threatening shocks [3, 11]. The symptoms span across days and sometimes weeks after the contact.

It is essential to highlight that no specific medication is designed for treating *T. processionea* envenomation. Antihistamines, commonly employed for allergic reactions, exhibit limited effectiveness in addressing the symptoms caused by the *T. processionea* venom [1]. Unfortunately, there is an escalating trend in reported health cases. These numbers are expected to keep rising in the upcoming years as a result of global warming favoring their survival and distribution [2, 12]. The growing prevalence of *T. processionea* in Western Europe drives the demand for an effective treatment to transform the innovative landscape by curing people. However, to develop an effective treatment, it is necessary to understand the role of the venom components in provoking the symptoms and unraveling the mechanism of action. Nature has fine-tuned the molecular diversity of venom components to be selective and effective on molecular targets such as ion channels and receptors. This could mean that possible modulators of ion channels or receptors present in the venom of *T. processionea* are causing the symptoms upon envenomation. However, the impact of *T. processionea* venom on ion channels and receptors related to itch and inflammation has not been explored. Unraveling the mechanism of action will improve the current state-of-the-art and pave the way to designing more effective treatment options. Moreover, it might be insightful to treat other caterpillar envenomations with a similar defense system worldwide.

## Materials and methods

### Collection and venom extraction from setae of *T. processionea*

In June and July 2020, larval colonies of *T. processionea* were gathered from oak (*Quercus*) trees in Mol (Limburg, Belgium). Larvae at the L5-L6 instar stage were individually extracted from their tents within a biosafety cabinet and placed in falcon tubes. Utilizing liquid nitrogen at −196 °C, the larvae were frozen, and subsequent shaking facilitated the release of urticating setae. Approximately 200 million setae were collected from 200 larvae and stored at −80 °C. The setae were dissolved in a 50% acetonitrile (ACN) solution, stirred for 4 h with a magnetic stirrer, and subjected to sonification at 30.0 Hz for 15 min. The resulting solution underwent clarification through centrifugation at 12,500 rpm for 10 min using an Eppendorf centrifuge 5810R (Eppendorf AG, Germany). The supernatant obtained was then subjected to lyophilization, yielding a desiccated substance for subsequent analytical investigations.

### Sample preparation for quadrupole time-of-flight mass spectrometry (Q-TOF–MS)

*T. processionea* venom extract was analyzed using positive-electrospray ionization (ESI) on a Bruker Impact II Q-TOF mass spectrometer (Bruker Daltonics, Germany). The venom extract was initially dissolved in ddH_2_O to achieve a 10 µg/µL stock concentration. A 1/10 dilution was prepared by dissolving 1 mg/mL *T. processionea* venom extract in 20% ACN followed by centrifugation. As a control, histamine and putrescine were purchased (Sigma Aldrich, USA), and various concentrations (0.1, 1, 10, and 100 μM) were prepared following the same protocol for quantification [13]. The Q-TOF system operated in positive mode with Bruker TargetScreener HR 4.0, allowing chromatographic separation and accurate mass detection within a 20-min analysis time. MS and MS/MS full scan mode datasets were collected and the raw MS data were formatted using Bruker Compass Data Analysis Viewer version 5.2 (Bruker Daltonics, Germany).

### Activity guided-purification via size-exclusion chromatography (SEC) and reversed phase C18-HPLC (RP-HPLC)

A total amount of 44 mg freeze-dried crude venom of *T. processionea* was further analyzed via size-exclusion chromatography (SEC) and fractions were collected from several runs. The venom sample was applied onto the Superdex 30 Increase 10/300 GL system, and separation was done at a flow rate of 1 mL/min using an isocratic gradient of 30% acetonitrile (ACN; vol/vol) in 0.1% trifluoroacetic acid (TFA; vol/vol) as eluent. Fractions obtained from SEC were collected and further separated by reversed-phase HPLC using a Shim-pack Arata C18 column with dimensions of 4.6  × 250 mm and particle size of 5 µM with a linear gradient from 0% to 80% of solution B (0.1% TFA in ACN, vol/vol) in solution A (0.1% TFA in water, vol/vol) at 1 mL/min over 80 min. The absorbance was monitored at wavelengths of 214 and 280 nm. Eluting compounds were collected in fractions and lyophilized. Each fraction was then evaluated for activity in the electrophysiological bio-assays. Active fractions underwent further characterization through a combined proteotranscriptomic pipeline.

### Proteotranscriptomic pipeline

#### Transcriptomics

For the transcriptome, RNA from one late-instar caterpillar was extracted with Qiagen RNeasy Micro kit (Qiagen, USA) (gut and head were removed) and sequenced via a dual-method approach, encompassing Single Molecule Real-Time (SMRT) Iso-Seq (Pacific Biosciences, United Kingdom) for highly accurate long reads and Illumina NovaSeq for precise short paired-end reads. A minimum of ≥ 300 ng of high-quality total RNA (NanoDrop ND-1000 UV/Vis instrument, USA) was utilized, meeting the criteria of an A260/280 ratio ~ 2.0, A260/230 ratio ≥ 2.0, and RNA integrity number ≥ 8.0 (Agilent 2100 Bioanalyzer, USA). For PacBio Iso-Seq, library preparation followed the Pacific Biosciences Iso-Seq™ Express Template Preparation protocol for Sequel II Systems. Sequencing was performed on a Pacific Sequel II instrument using Binding kit 2.0 and Sequencing Chemistry v2.0, utilizing a single SMRT cell. All procedures, including library preparation, sequencing, HiFi read generation, and IsoSeq processing, were conducted at the Norwegian Sequencing Centre PacBio node, situated at the University of Oslo Department of Biosciences in Oslo, Norway. Concerning the Illumina NovaSeq platform, TruSeq libraries featuring 150 bp inserts were prepared and subjected to paired-end sequencing following the manufacturer's guidelines. After sequencing, reads were checked for quality and length distribution with FastQC v11.9 [14]. Trimmomatic v0.39 [15] was used to trim TruSeq3-PE adapters and remove low-quality reads. Quality trimming used a 4-base sliding window and a minimum quality threshold of 20, discarding sequences shorter than 60 bases post-trimming. These library preparation and sequencing steps took place at the Norwegian Sequencing Centre Illumina node within the Oslo University Hospital, Oslo, Norway. The transcriptome was generated through short reads assembled with the long-reads as reference using Trinity v2.10 [16]. TransDecoder v5.5.0 [17] was applied to extract open reading frames (ORFs) encoding 50 amino acids or more to create an amino acid sequence database for automated MS data searches in proteomics. A detailed description and analysis of the transcriptome can be found in part 1.

#### Proteomics of active venom fractions from *T. processionea*

Proteomic data were acquired by analyzing active venom fractions with a trapped ion mobility spectrometry on a timsTOF Pro mass spectrometer (Bruker, USA). Three distinct samples were prepared for timsTOF analysis: native (top-down), reduced and alkylated (RA) (top-down), and RA digested (RAT) (bottom-up). Native samples were dissolved in a solution containing 5% acetonitrile (ACN) and 1% formic acid. Reduced and alkylated samples underwent reduction with 5 mM dithiothreitol in 50 mM ammonium bicarbonate and 5% ACN at 65 °C for 5 min, followed by alkylation with 10 mM iodoacetamine in 50 mM ammonium bicarbonate and 5% ACN at 30 °C for 30 min. RA digested samples, after reduction and alkylation, were digested overnight with trypsin (42 ng/µL in 50 mM ammonium bicarbonate and 5% ACN at 37 °C). Desalting using a C18 ZipTip (Thermo Fisher, USA) and re-dissolving in 1% formic acid preceded analysis on a timsTOF Pro mass spectrometer (Bruker, USA). The resulting mass spectrometry datasets (untreated—N, reduced and alkylated—RA, and reduced, alkylated, and trypsin-digested—RAT) were searched against translated predicted ORFs from the transcriptome of *T. processionea* and a list of 23 common contaminants via Peaks Studio v8.5. A local FDR of < 0.1% was considered significant for peptide/protein identification. Peptide matches were required to have a minimum −10lgP score of 15 (*p* ≤ 0.03).

### Peptide synthesizes

A venom peptide, TPTX_1_-Tp1, was synthetically designed using Fmoc (*N*-(9-fluorenyl) methoxycarbonyl) solid-phase peptide synthesis chemistry by GenicBio Limited (Shanghai, China). The synthesized peptide was lyophilized and kept at room temperature (18–22 °C). The peptide was folded by dissolving it in a physiological buffer solution ND96 (96 mM NaCl, 2 mM KCl, 2 mM MgCl_2_, 1.8 mM CaCl_2_ and 5 mM HEPES) at a concentration of 1 mg/mL. A quality check was performed with RP-HPLC.

### *Xenopus laevis* frogs and the isolation of oocytes by partial ovariectomy

All experiments and procedures involving *Xenopus laevis* frogs throughout this study received approval from the Ethical Committee for Animal Research at KU Leuven (Project No. P186/2019 and No. P074/2023). The research adhered to the guidelines outlined in Directive 210/63/EU of the European Union regarding the welfare of laboratory animals. Before harvesting stage V–VI oocytes from ovarian tissue, adult female *Xenopus laevis* frogs underwent immersion in an aqueous solution containing 0.1% buffered tricaine (ethyl 3-aminobenzoate methanesulfonate, 1 g/L, Sigma-Aldrich, USA) and NaHCO_3_ (sodium bicarbonate, 1 g/L; Sigma-Aldrich, USA) in aquarium water (pH 7.5) for a duration of 15 min. Following the recovery period, the frogs were monitored daily and returned to their tanks at the Aquatic Facility of KU Leuven. The surgically removed ovarian lobes underwent enzymatic defolliculation in a Ca^2+^-free ND96 solution (96 mM NaCl, 2 mM KCl, 2 mM MgCl_2_, and 5 mM HEPES), supplemented with collagenase from *Clostridium histolyticum* type IA, 1.5 mg/mL; Sigma-Aldrich, USA) on a rocker platform at 16 °C for 2.5 h. Following enzymatic defolliculation, the oocytes were transferred to a calcium-containing ND96 buffer (96 mM NaCl, 2 mM MgCl_2_, 2 mM KCl, 5 mM HEPES, and 1.8 mM CaCl_2_; pH 7.5) supplemented with gentamicin (100 mg/L; Schering-Plough, Belgium) and theophylline (90 mg/L; ABC chemicals, Belgium) at 16 °C.

### In vitro transcription of cDNA clones

We used *Xenopus laevis* oocytes as a heterologous expression system in electrophysiological experiments to express ligand-gated and voltage-gated ion channels. Molecular biology techniques (subcloning, transformation, linearization and transcription) were used to make RNA encoding: (1) transient receptor potential channel (hTRPV1 and hTRPA1), (2) histamine receptors (hH1R and hH4R), (3) bradykinin receptor (hB2R), (4) nicotinic acetylcholine receptor (adult muscle type hα1β1δε nAChR and neuronal hα7 nAChR), (5) acid-sensing ion channel (rASIC1a), (6) voltage-gated sodium channels (rNa_v_1.1, rNa_v_1.2, rNa_v_1.3, rNa_v_1.4, mNa_v_1.6 and hNa_v_1.8) and their auxiliary subunits rβ1 and hβ1 and (7) potassium channels (hK_v_1.1, hK_v_1.2, hK_v_1.3, hK_v_1.4, hK_v_1.5, hK_v_1.6, hK_v_3.1, hK_v_10.1, mGIRK1, mGIRK2 and mIRK1). Plasmids corresponding to each channel or receptor underwent linearization using specific restriction enzymes and subsequent transcription with the T3, T7, or SP6 mMESSAGE mMACHINE transcription kit (Ambion, USA). Depending on the channel type, defolliculated oocytes were injected with 10–50 nL of cRNA at a concentration of 1 ng/nL using a micro-injector (Nanoject II; Drummond Scientific Company, USA). Electrophysiological experiments were conducted following cRNA injection, with an incubation period of 1–5 days at 16 °C in ND96 buffer.

### Electrophysiological recordings with a two-electrode voltage clamp (TEVC)

A two-electrode voltage clamp (TEVC) system utilizing a GeneClamp 500 amplifier (Molecular Devices, Downingtown, PA, USA) was under the control of a pClamp data acquisition system (Axon Instruments, USA), and pClamp Clampex 10.4 software (Axon Instruments®, USA). This setup was employed to measure currents across the cell membrane. The whole-cell currents from the oocytes were recorded at room temperature (18–22 °C). Two micro-electrodes, comprising voltage and current electrodes, were crafted from borosilicate glass capillaries (1.14 mm outside diameter, 0.7 mm inside diameter), pulled using a microelectrode puller, PUL-1 (World Precision Instruments, USA). These electrodes were filled with 3 M KCl using a MicroFil needle, and their resistance was maintained between 0.5 and 1.5 MΩ. Throughout the measurements, oocytes were positioned in a 200 µL recording chamber. A membrane test was initially conducted to adjust measurement parameters based on the membrane quality. For ligand-gated ion channels and receptors, a control response with an agonist was performed to check the expression of the channel. After expression check in voltage- and ligand-gated ion channel and receptor experiments, the sample was applied until a stable activated or blocked state of the channel was reached (when the agonist/antagonist activity was visible) or at least 1 min when no significant change in current was visible. In all experiments, 80 µg of crude venom extract from *T. processionea* was directly pipetted into a 200 µL bath, resulting in a final concentration of 0.4 µg/µL tested. Fractions obtained after SEC and RP-HPLC were dissolved in 100 µL of ND96 solution and tested by pipetting 5 µL into a 200 µL bath. Several concentrations of the synthetic peptide (TPTX_1_-Tp1) were tested. After sample application, the agonist was applied again to test reversibility in ligand-gated ion channel/receptor experiments.

TRPV1 and TRPA1 currents were continuously monitored in a perfusing ND96 solution, following a protocol at −90 mV for 400 s. The sampling rate was set at 100 Hz, and the signals were filtered at 20 Hz. A 2-s voltage ramp protocol from −120 to + 70 mV, initiated from a holding potential of −20 mV, was applied to investigate the voltage dependency. 0.1 or 1 µM capsaicin (Sigma-Aldrich, USA) and 10 µM capsazepine (Sigma-Aldrich, USA) were used for TRPV1. In the TRPA1 experiments, 50 µM mustard oil was used.

The H1R and B2R couples with Gα_q/11_ proteins, initiating the release of Ca^2+^ from the intracellular stores. This rise in intracellular Ca^2+^ will activate the Ca^2+^-activated Cl^−^ channels, serving as a sensitive readout. The currents induced by 1 µM histamine (Sigma-Aldrich, USA) or 1 µM bradykinin (Sigma-Aldrich, USA) were sampled at 1000 Hz, filtered at 20 Hz, and assessed through a 2-s voltage ramp protocol spanning from −120 to + 70 mV, initiated from a holding potential of −20 mV during perfusion. A detailed description of the electrophysiological experiments of H1R and analog for B2R can be found in Seldeslachts et al. [18].

To conduct GIRK1/2 and IRK1 measurements, oocytes were subjected to voltage clamping at −90 mV, and the currents were recorded by transitioning from a low-potassium ND96 solution to a high-potassium solution (HK; 96 mM KCl, 2 mM NaCl, 1 mM MgCl_2_, 1.8 mM CaCl_2_ and, 5 mM HEPES with a final pH of 7.5). The recorded currents were filtered at 20 Hz and sampled at 100 Hz.

The H4R was coupled to the inward rectifier potassium channels (GIRK1 and GIRK2) and RGS4 via Gα_i/o_. The first increase in inward K^+^ currents was induced by a HK solution, signifying a ‘basal’ K^+^ current (*I*_K_,_basal_), indicating receptor-independent GIRK channel activation. In the presence of an agonist, 1 µM histamine in HK (Sigma-Aldrich, USA), this *I*_K_,_basal_ was enhanced, representing the histamine-induced *I*_K_,_histamine_. The histamine-induced current is reversible after histamine washout with HK. A concentration of 24 nM JNJ 7777120 (Sigma-Aldrich, USA) was used as a potent H4R antagonist. During the experiments involving pertussis toxin (PTX) (Sigma-Aldrich, USA), oocytes were immersed in ND96 solution supplemented with 2.5 ng of PTX for 16 h. This incubation period was implemented to assess the inhibitory impact of PTX on G protein-coupled receptor (GPCR) signaling before conducting measurements. Oocytes were voltage clamped at −90 mV, and currents were sampled at 100 Hz and filtered at 20 Hz. A detailed description of the electrophysiological experiment of the coupling of H4R with GIRK1, GIRK2, and RGS4 can be found in Seldeslachts et al. [18].

For muscle-type α1β1δε and neuronal-type α7 nAChR, oocytes were clamped at a holding potential of −70 mV, sampled at 100 Hz, and filtered at 20 Hz [19]. The same protocol was employed for ASIC1a channel measurements. Currents were elicited with 100 µM acetylcholine (ACh) for the nAChR experiments and by exchanging ND96 solution at pH 7.5 with ND96 solution at pH 4.5 for the ASIC1a experiments.

For voltage-gated sodium and potassium channel protocols, the recorded currents were sampled at specific rates: 20 kHz for Na_v_1.x, 10 kHz for K_v_1.x, hERG, and Shaker-IR. Via a four-pole low-pass Bessel filter, the currents were filtered at 2 kHz for Na_v_1.x, 500 MHz for K_v_1.x, and 1 kHz for hERG and Shaker-IR. Leak subtraction was performed using a −P/4 protocol. Na_v_1.x traces were generated through 100 ms depolarizations to the voltage corresponding to maximal Na^+^ current under control conditions (*V*_max_). Current–voltage relationships were examined using 50 ms step depolarizations ranging from −90 to + 40 mV with 5 mV increments. A two-step protocol was utilized for inactivation, involving a 100 ms pulse from −90 to 0 mV with a 5 mV step, followed immediately by a test pulse to 0 mV. Subsequently, K_v_1.x and Shaker-IR current traces were elicited by 500 ms depolarization pulses to 0 mV, followed by 500 ms repolarization pulses to −50 mV. hERG1 current traces were induced by applying + 40 mV pre-pulses for 200 ms, immediately followed by a step of −120 mV for 200 ms.

### Calcium imaging

HEK-hTRPM3 were plated on the day before the measurement in 96-well plates at 100,000 cells/well [20]. Before measurement, cells were loaded with FURA-2-AM for 30 min at 37 °C, washed and 100 μL of a solution containing (in mM) 150 NaCl, 6 KCl, 1.5 MgCl_2_, 2 CaCl_2_, 10 glucose and 10 HEPES (pH 7.4 with NaOH) was added to each well. HEK-mTRPV2 cells were seeded at a density of 50,000 cells/well in a 96-well plate [21]. Cells were incubated with 2 μM Fura-2 acetoxymethyl ester (Fura-2 AM) for 60 min at 37 °C. Measurements were performed in a standard solution containing (in mM) 150 NaCl, 2 CaCl_2_, 1 MgCl_2_, 10 d-glucose and 10 HEPES (pH 7.4 with NaOH). To simultaneously screen for potential agonists and antagonists, venom extract was applied to tetrahydrocannabinol (THC) supplementation (50 μM, TRPV2) or pregenolone sulfate (PS) supplementation (40 µM, TRPM3). Fluorescence signals were recorded before, during and after the addition of THC at excitation wavelengths of 340 and 380 nm. The experiment was considered successful when the THC (TRPV2) or PS (TRPM3) response after vehicle application exceeded a minimal F340/380 increase of 0.5.

### Data and statistical analysis of the electrophysiological experiments

Electrophysiological data were acquired through pClamp Clampex 10.4 (Axon Instruments, USA), subjected to analysis using pClamp Clampfit 10.4 (Axon Instruments, USA), and presented as the means ± standard error of the mean (SEM) from *n* ≥ 3 independent experiments. Concentration–response curve was constructed employing Origin 9.0 software (Origin Lab, USA). Given its capability to model sigmoidal relationships typically observed in the concentration–response curves, the four-parameter Hill equation was used for data fitting. The Hill coefficient was permitted to vary, allowing flexibility to account for potential changes in cooperativity or interaction. The utilized Eq. (1) is expressed as follows:

1 $$y=A1+\frac{A2-A1}{1+{10}^{\left(Logx0-x\right)p}}$$

*A1* bottom asymptote; *A2* top asymptote; *Logx0* center; *P* hill slope.

Statistical analyses were conducted to assess the goodness-of-fit of the concentration–response curves. The reduced chi-squared and adjusted R-squared were employed as performance criteria for model selection. An ideal fit for the reduced chi-squared is typically close to 1. A higher value for the adjusted R-squared, closer to 1, signifies a good fit. The calculated EC_50_ value is presented as the mean ± standard error of the mean (SEM). The concentration–response curve was plotted as a percentage of activation against the logarithm of the various concentrations tested. The transformation of concentrations aimed to stabilize the variance across the concentration range, ensuring a more consistent spread of biological responses to meet the homogeneity of variance. All experiments were replicated at least three times (*n* ≥ 3). GraphPad Prism (version 8.1.2, GraphPad Software, USA) was employed for statistical analysis via multiple *t*-test with statistical significance determined using the Holm–Sidak method, with *p* ≤ 0.05, for the Na_v_ and K_v_ channel experiments. Statistical significance levels are denoted as follows: *p < 0.05, **p < 0.01, ***p < 0.001, ****p < 0.0001.

## Results

### Unveiling the role of TRPV1: investigating the effects of setae whole venom extract in bioassays

Itch and inflammation represent pivotal events in the pathogenesis of *T. processionea* envenomation. To identify the molecule(s) in *T. processionea* venom that trigger these events, we looked into how various molecular targets in the pathway are involved. Consequently, a setae extract was prepared and tested for activity in functional studies on hTRPV1, hTRPA1, hH1R, hH4R, hB2R, hα1β1δε nAChR, hα7 nAChR, rASIC1, rNa_v_1.1, rNa_v_1.2, rNa_v_1.3, rNa_v_1.4, mNa_v_1.6, hNa_v_1.8, hK_v_1.1, hK_v_1.2, hK_v_1.3, hK_v_1.4, hK_v_1.5, hK_v_1.6, hK_v_3.1, hK_v_10.1, mGIRK1, mGIRK2 and mIRK1.

**Fig. 2:**
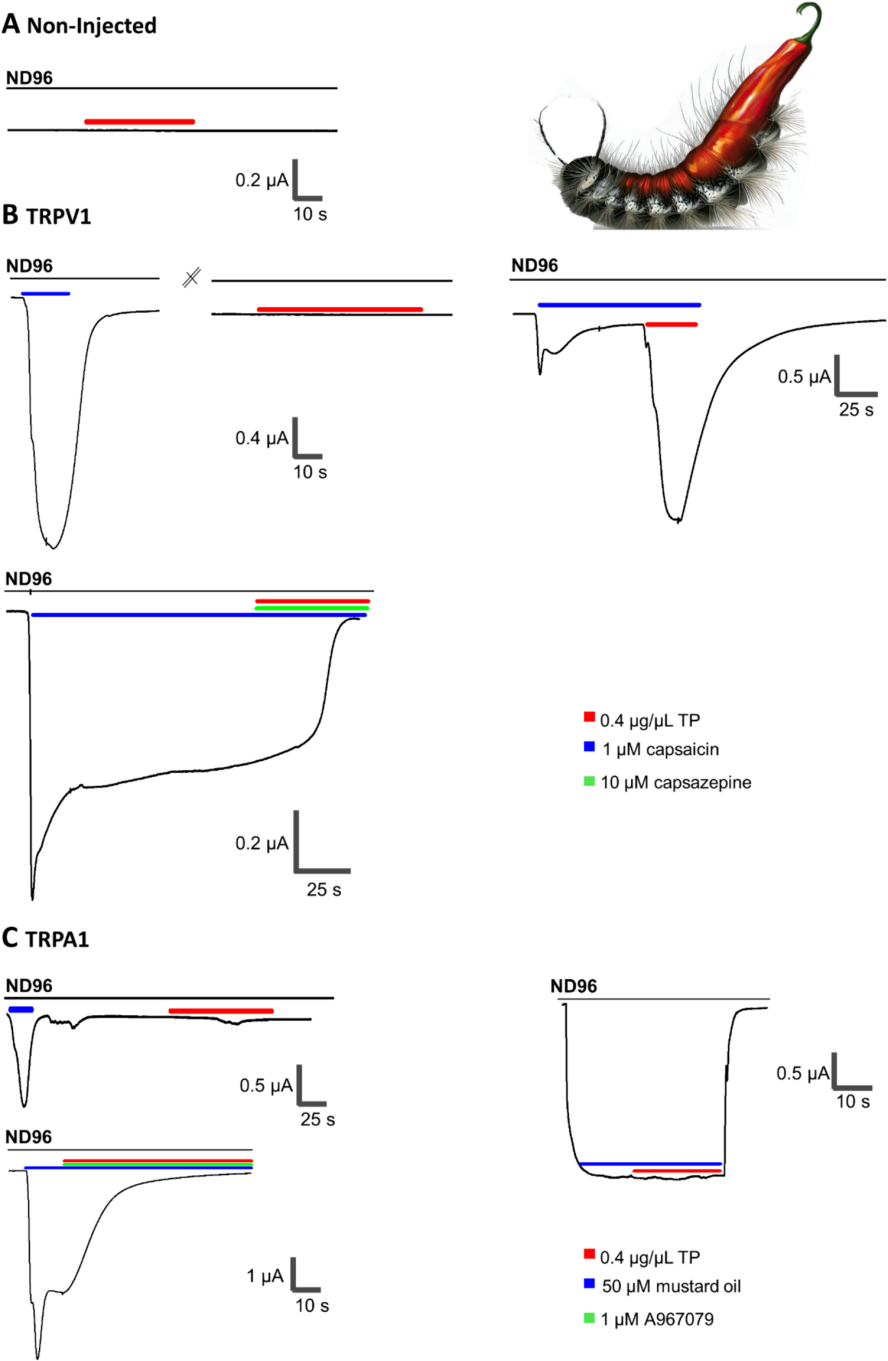
Effect by crude *Thaumetopoea processionea* venom extract on non-injected, hTRPV1 and hTRPA1 injected *Xenopus laevis* oocytes. **A** A representative validation curve measured with a holding potential of −90 mV shows no appreciable current enhancement by *T. processionea* venom extract (red) in non-injected *Xenopus laevis* oocytes. **B** Three representative traces measured with a holding potential of −90 mV. Left: A representative validation trace shows a current increase in the presence of 1 µM capsaicin (blue) in TRPV1-injected *Xenopus laevis* oocytes. No effect of TRPV1 expressing oocytes incubated with 0.4 µg/µL crude *T. processionea* venom setae extract (red). Right: Current increase by the application of 1 µM capsaicin (blue) and by 0.4 µg/µL *T. processionea* venom setae extract (red) in a solution with 1 µM capsaicin (blue). Below: Effect of 0.4 µg/µL *T. processionea* venom setae extract (red) and 1 µM capsaicin (blue) is blocked by the addition of 10 µM capsazepine (green). **C** Three representative traces were measured with a holding potential of −90 mV. Left: A representative validation trace shows a current increase in the presence of 50 µM mustard oil (blue) in TRPA-injected *Xenopus laevis* oocytes. No appreciable current enhancement by 0.4 µg/µL *T. processionea* whole venom setae extract (red). Right: Current increase by the application of 50 µM mustard oil (blue) but not by 0.4 µg/µL *T. processionea* venom setae extract (red) in a solution with 50 µM mustard oil (blue). Below: Effect of 50 µM mustard oil (blue) is blocked by the addition of 1 µM A967079 (green). All experiments were replicated at least three times (*n* ≥ 3)

The crude venom extract of *T. processionea* venom was first tested on non-injected oocytes. Figure 2A clearly shows that 0.4 µg/µL venom extract does not change the membrane current on non-injected oocytes and thus does not directly activate endogenous ion channels/receptors expressed on the cell membrane of *Xenopus laevis* oocytes.

Following the non-injected oocytes, the activity on hTRPV1, mTRPV2, hTRPM3, and hTRPA1 receptors was tested. On TRPV1-injected oocytes, no significant effect was seen by the venom on its own (Fig. 2B, left). However, intriguingly, when co-applied with the agonist, 1 µM capsaicin, 0.4 µg/µL whole venom setae extract was able to potentiate TRPV1 (Fig. 2B, right). The pronounced inward current is a result of the influx of cations, leading to cellular depolarization. This depolarization event has the potential to stimulate nociceptive nerve terminals, subsequently triggering an increase in the release of calcium ions and substance P, which is implicated in inflammation, itch, and pain. More importantly, the current increase by whole venom setae extract disappeared with the addition of an antagonist, 10 µM capsazepine (Fig. 2B, below). Interestingly, the whole venom setae extract of *T. processionea* does not modulate the TRPV2, TRPM3 and TRPA1 channel activity (Fig. 2C and Supplementary Fig. S1). Furthermore, experiments were conducted to assess the effect of co-applying whole venom extract and 50 µM mustard oil on TRPA1. However, unlike TRPV1, this combination did not elicit a significant increase in current (Fig. 2C). It can be concluded that, for the first time, we indicated TRPV1 as an important molecular target in the envenomation of *T. processionea*.

**Fig. 3:**
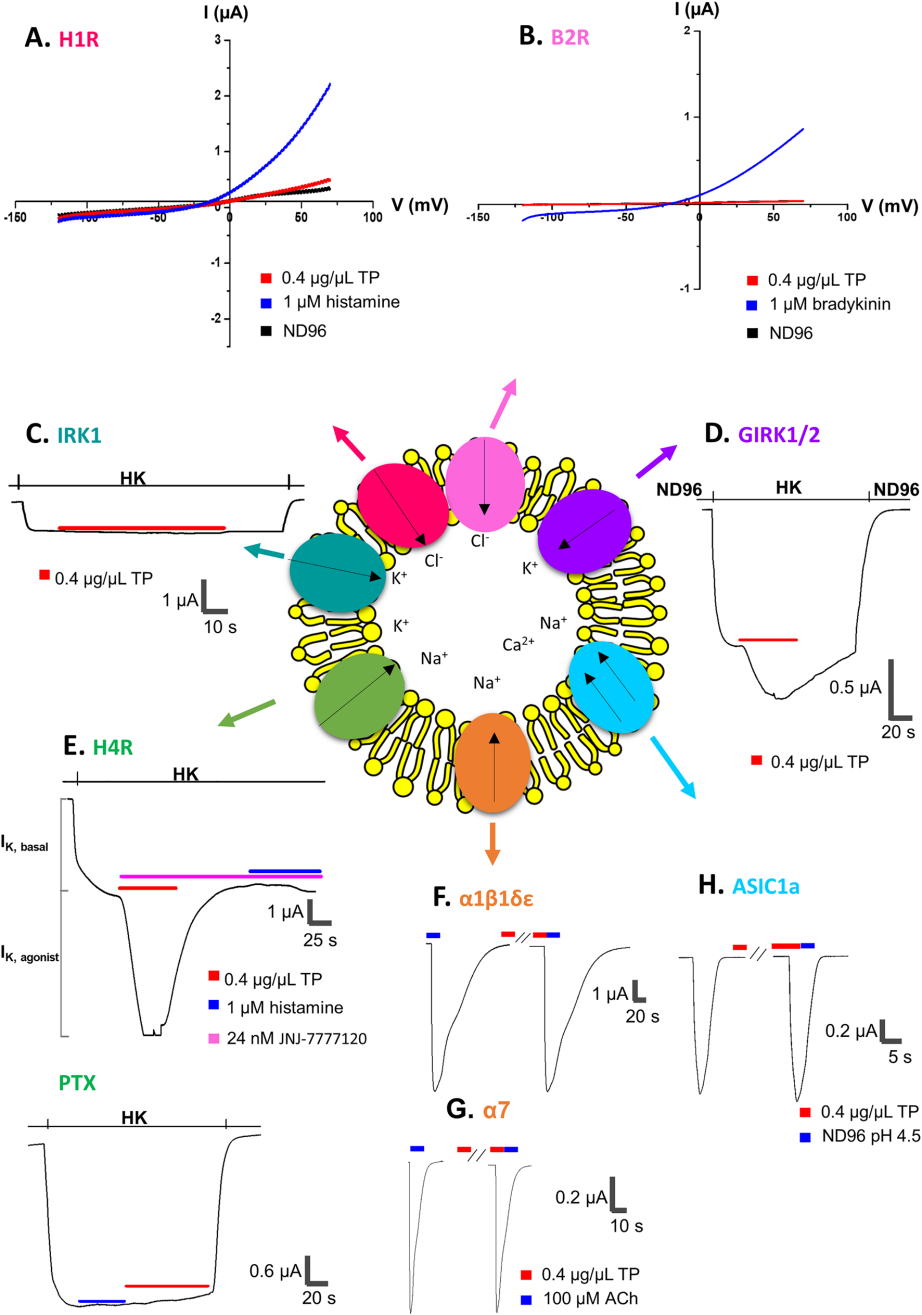
Effect by crude *Thaumetopoea processionea* venom setae extract on H1R, B2R, IRK, GIRK1/2, H4R-GIRK1/2-RGS4, α1β1γδ, α7 and ASIC1a injected *Xenopus laevis* oocytes.** A** A representative trace of H1R measured with a ramp protocol from −120 to + 70 mV from a holding potential of −20 mV shows a current increase upon application of 1 µM histamine (blue) and no significant change in current by 0.4 µg/µL *T. processionea* venom setae extract (red). **B** A representative trace of 1 µM bradykinin measured with a ramp protocol from −120 to + 70 mV from a holding potential of −20 mV evoked a change of conductance (blue), and no significant trace was evoked by 0.4 µg/µL *T. processionea* venom setae extract on B2R injected oocytes. **C** No alteration of the current was observed after the addition of 0.4 µg/µL *T. processionea* venom setae extract to IRK1 measured with a holding potential of −90 mV. **D** A representative trace measured with a holding potential of −90 mV shows a current enhancement by the addition of 0.4 µg/µL *T. processionea* venom setae extract (red) to GIRK1/2 injected oocytes. **E** A representative trace of H4R measured with a holding potential of −90 mV shows that the effect of *T. processionea* venom setae extract (red) could not be blocked in the presence of the JNJ 7777120 antagonist (pink). The effect of histamine (blue) was blocked. No alteration of the current was observed after the addition of 0.4 µg/µL *T. processionea* venom setae extract (red) to H4R co-injected GIRK1/2 RGS4 oocytes after treatment with PTX. **F**, **G** Electrophysiological profile of muscle-type α1β1γδ nAChR and neuronal-type of a7 nAChR measured at a holding potential of −70 mV. The nAChRs were gated by 100 μM ACh (blue) at 1 mL/min. The first and the second peak amplitude represent the absence and presence of 0.4 μg/μL of *T. processionea* venom. **H** Whole-cell ASIC1a current traces measured at holding potential of −70 mV in control pH 4.5 and in the presence of *T. processionea* venom setae extract in pH 7.5 and pH 4.5

Furthermore, our investigations revealed that the *T. processionea* whole venom setae extract does not elicit a significant change in conductance in oocytes expressing the H1R and B2R (Fig. 3A, B). Next, the activity of *T. processionea* whole venom setae extract was tested for activity on GIRK1/2 and IRK channels.

In these experiments, 0.4 µg/µL whole venom setae extract of *T. processionea* (red) could not show an enhanced activity on IRK injected oocytes (Fig. 3C) while it enhanced the inward K^+^ current in GIRK injected oocytes (Fig. 3D).

IRK channels differ from GIRK channels mainly because they do not interact with G proteins. Additionally, we saw an enhancement of inward K^+^ current on H4R-GIRK1/GIRK2-RGS4-injected oocytes, even when all H4R receptors in the same cell were antagonized by 24 nM JNJ 7777120 (pink), a specific H4R antagonist with an IC_50_ of 5 ± 0 nM (Fig. 3E). More interestingly, the effect disappears when the H4R-GPCR signaling is blocked with a G protein blocker, PTX (Fig. 3E). From these results we can conclude that the venom does not activate the H4R and GIRK1/2 directly but probably plays a role in the GPCR signaling of an endogenous expressed GPCR in *Xenopus laevis* oocytes. In this research, we could not identify this endogenous GPCR.

In the subsequent analysis, we probed the pharmacological profile and impact of *T. processionea* whole venom extract on oocytes expressing the muscle type of α1β1δε nAChR (Fig. 3F) and the neuronal type of α7 nAChR (Fig. 3G). Intriguingly, there was no discernible alteration in current amplitude following the application of 0.4 µg/µL *T. processionea* venom extract. Subsequently, the influence of *T. processionea* whole venom was assessed on ASIC1a channels (Fig. 3H). At both pH 7.5 and pH 4.5, no substantial modulation in current was evident upon applying 0.4 µg/µL venom extract.

*T. processionea* venom activity was also screened against Na_v_1.1, Na_v_1.2, Na_v_1.3, Na_v_1.4, Na_v_1.6, and Na_v_1.8 (Supplementary Fig. S2). For Na_v_1.1, a small but significant decrease in current was observed between −15 and −5 mV (*p* < 0.0001, *n* ≥ 3). Similarly, we observed a small but significant decrease in current for Na_v_1.4 between −10 to 10 mV (****p < 0.0001 for −10, −5 and 10 mV; ***p < 0.001 for 0 and 5 mV, *n* ≥ 3) and for Na_v_1.8 between 5 to 40 mV (****p < 0.0001 for 10–35 mV; ***p < 0.001 for 10 and 40 mV, *n* ≥ 3). No significant modulation of the gating kinetics or voltage-dependence of activation and steady-state inactivation was observed for any of these channels. Also, no significant modulation of the voltage-dependence of activation nor steady-state inactivation was observed for Na_v_1.2, Na_v_1.3, or Na_v_1.6.

Subsequently, *T. processionea* venom extract was tested for its activity against K_v_1.1–K_v_1.6, K_v_10.1, and K_v_3.1 as shown in Supplementary Fig. S3. At a concentration of 0.4 µg/µL, *T. processionea* venom has a small but significant effect on K_v_1.2 (**p < 0.01 for −30 to 20 mV and 60 mV; *** *p* < 0.01 for 30–50 mV, *n* ≥ 3), K_v_1.3 (**p < 0.01 for −10 mV; *** *p* < 0.01 for 20–60 mV, *n* ≥ 3) and K_v_3.1 (**p < 0.01 for −10 and 60 mV; ***p < 0.01 for 0–50 mV, *n* ≥ 3). No effect was seen for K_v_1.1, K_v_1.4, K_v_1.5, K_v_1.6 and K_v_10.1.

### A secapin-like peptide is responsible for the TRPV1 modulation

To identify the molecule(s) in *T. processionea* venom responsible for the TRPV1 activity, we subjected the whole venom compound mixture to fractionation using SEC and RP-HPLC. The 62 obtained fractions were evaluated for activity on the TRPV1 channels in our electrophysiological bio-assay. After SEC, four early eluting fractions from the RP-HPLC induced TRPV1 activity when co-applied with 1 µM capsaicin. The other fractions tested did not show any significant effect on TRPV1.

**Fig. 4:**
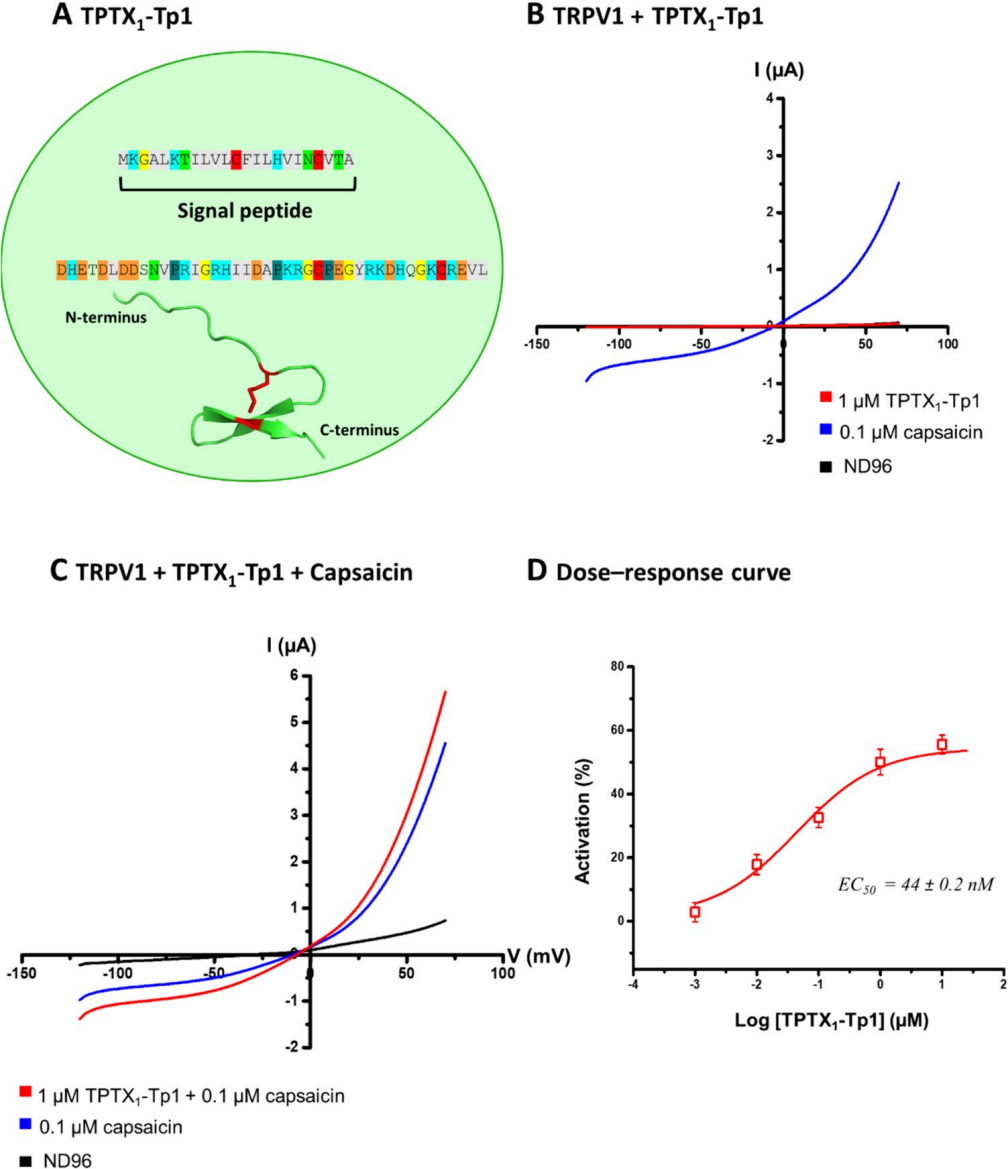
Electrophysiological characterization of TPTX_1_-Tp1 from *Thaumetopoea processionea* on human TRPV1.** A** Sequence and 3D structure predicted by AlphaFold2. Residues in gray represent amino acids with a hydrophobic side chain, residues in light blue represent a positively charged chain, negatively charged side chain in orange, glycine in yellow, proline in green, cysteine in red and amino acids with an uncharged polar side chain does not have a color. **B** A representative ramp protocol trace shows no appreciable current enhancement by 1 µM TPTX_1_-Tp1 (red) in TRPV1 expression *Xenopus laevis* oocytes. **C** A representative ramp protocol trace where TPTX_1_-Tp1 was able to cause an increase in current when dissolved in a solution together with 0.1 µM capsaicin. **D** A dose–response curve is presented with the percentage of activation plotted against the logarithmically scaled concentrations. The maximum current potentiation of TPTX_1_-Tp1 on top of 0.1 µM capsaicin at approximately 10 µM concentration (55%), and the calculated EC_50_ value was estimated at 44 ± 0.2 nM

The four fractions underwent further characterization through the proteotranscriptomic pipeline, and this revealed the presence of the seven secapin-like peptides (Supplementary Information about secapin-like peptides in *T. processionea* and Supplementary Fig. S4). Of interest, TPTX_1_-Tp1 displayed in Fig. 4A.

To validate the expected pharmacological activity, TPTX_1_-Tp1 was synthesized chemically and tested for activity on TRPV1 injected oocytes using the electrophysiological bio-assay. These electrophysiological experiments were recorded with a ramp protocol from −120 to + 70 mV from a holding potential of −20 mV in ND96. It is noticeable that TPTX_1_-Tp1 alone does not have an effect on TRPV1 injected oocytes (Fig. 4B). Nevertheless, when combined in a solution with 0.1 µM capsaicin, it induces an increase in the current (Fig. 4C). We observed a maximum current activation in the presence of 0.1 µM capsaicin at approximately 10 µM (55% potentiation), and an EC_50_ value of 44 ± 0.2 nM was determined (Fig. 4D). A potential concentration depended effect of capsaicin has been investigated but did not allow us to draw further conclusions.

### Additional clues to TRPV1 modulation unveiled in venom: the role of putrescine in a venom without histamine

**Fig. 5:**
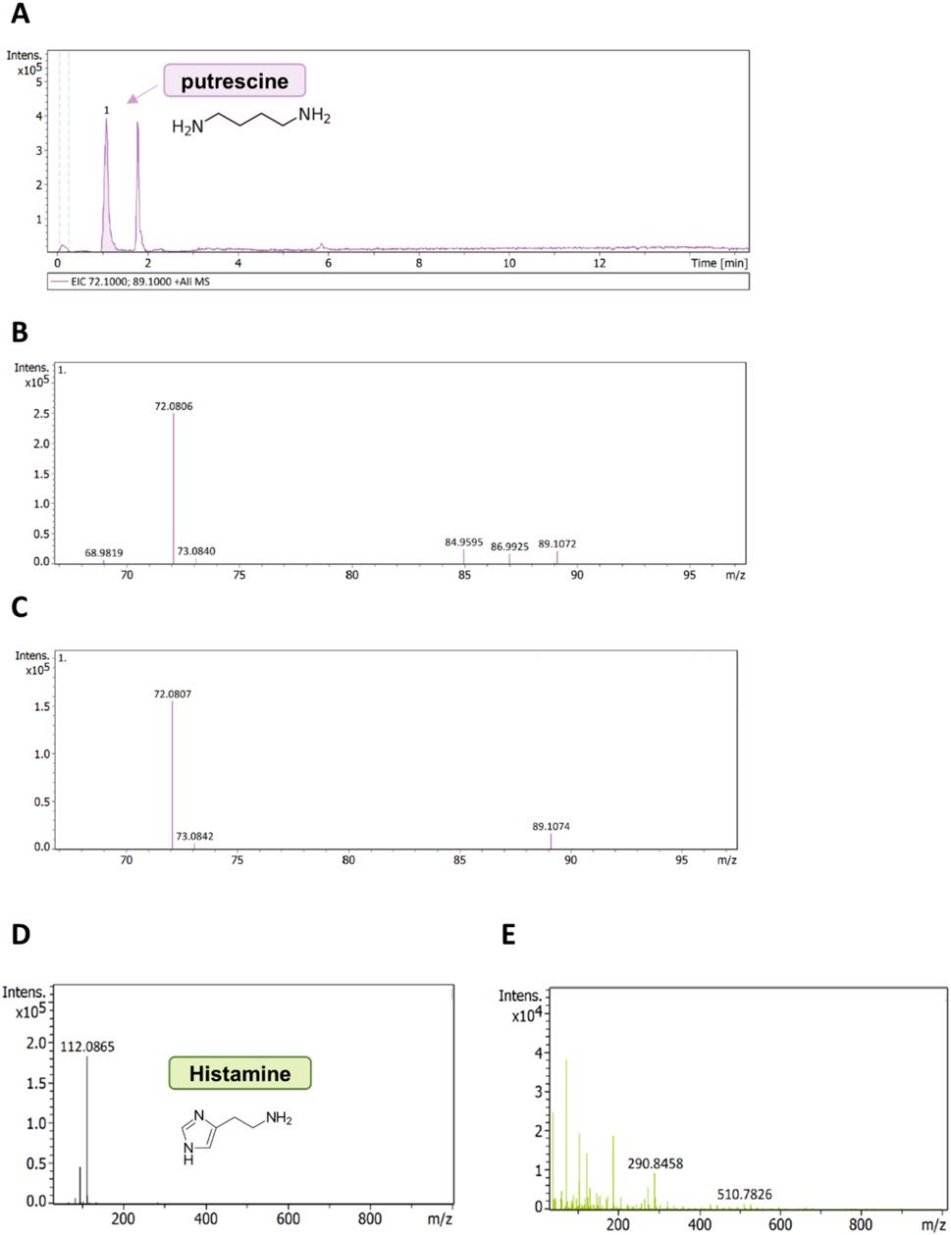
Discovery of putrescine and absence of histamine in the venom extract of *Thaumetopoea processionea* via Q-TOF–MS. **A** LC–MS chromatogram of 10 µg/µL *T. processionea* venom extract with the discovery of putrescine (retention time: 1.1). **B** MS/MS spectra of 10 µg/µL *T. processionea* venom extract with *m*/*z* signals of the precursor ion [M + H]^+^ 89.1 and fragmentation product ion *m*/*z* 72.1 and *m*/*z* 73.1. **C** MS/MS spectra of 100 µM putrescine with *m*/*z* signals of the precursor ion [M + H]^+^ 89.1 and the fragmentation product ion *m*/*z* 72.1 and *m*/*z* 73.1. **D** MS spectra of 10 µM histamine with *m*/*z* signals of [M + H]^+^ 112.1. **E** Absence of histamine in MS spectra of 10 µg/µL *T. processionea* venom

Following our thorough screening and the discovery of a peptide in the caterpillar venom influencing TRPV1, we have uncovered further indications suggesting the modulation of TRPV1. We analyzed the crude venom extract of *T. processionea* setae for small molecules and inflammatory mediators such as polyamines and histamine. The presence of putrescine in the venom extract was found via top-down proteomics using an ESI-Q-TOF mass spectrometer. Putrescine has a molecular formula of C_4_H_12_N_2_, a molecular weight of approximately 88.15 g/mol, a retention time of 1.1 min, and exists as a single charged ion [M + H]^+^ in positive ionization mode (Fig. 5A).

Figure 5B, C display the MS/MS spectra corresponding to 10 µg/µL *T. processionea* setae extract and 100 µM putrescine, respectively. In this MS/MS spectra, the *m*/*z* signal of the precursor ion was 89.1, aligning with the specific mass-to-charge ratio described in the literature and the theoretical spectra from 100 µM putrescine. The fragment ions with masses of 73.08 and 72.08 are likely the result of the loss of an amino group [M + H-NH_2_]^−^ and ammonia [M + H-NH_3_]^+^, respectively, from the precursor ion of putrescine resulting in the formation of smaller fragments. An effort was made to quantify the amount of putrescine in the venom extract. A calibration curve (*y* = 0.000032*x* − 5.5951) was established to achieve this, yielding a measurement of 74 µM putrescine in the venom extract with a concentration of 10 µg/µL.

A similar analysis was conducted to detect the presence of histamine (the same protocol was used as described by Seldeslachts et al. [13]). Figure 5D, E display the MS spectra corresponding to 10 µM histamine and 10 µg/µL *T. processionea* setae extract, respectively. No histamine was identified in 10 µg/µL *T. processionea* setae extract.

## Discussion

A distinctive array of symptoms unfolds in the intriguing onset of *T. processionea* envenomation, painting a unique clinical portrait. Between 6 to 8 h post-contact, an irresistible itch reaction surfaces, serving as a clear indicator of the initiation of the envenomation process. Remarkably, during this initial period, a visible redness is absent. The visible symptoms of local contact dermatitis only manifest later in the progression. What makes this phenomenon of delayed manifestation even more intriguing is the persistence of the itch over several days or weeks after the contact. These effects are believed to be triggered by the simultaneous action of two distinct mechanisms [12]. It is evident that the mechanical injury resulting from setae penetrating the skin can be accountable for local irritation. However, the prolonged effects and the fact that symptoms only manifest 6–8 h after contact indicate a synergy with components present in the venom stored within the hollow structure of the setae or present on the outside of the setae [2].

Our results indicate that TRPV1 is involved in the pathway through which *T. processionea* causes its pathophysiological effects. Earlier research has consistently underscored the role of TRPV1 in triggering itch across diverse physiological and pathological contexts [22–24]. Moreover, we have discovered a secapin-like peptide called TPTX_1_-Tp1, extracted from *T. processionea* venom, that is responsible for potentiating these TRPV1 channels. Research on these secapins or secapin-like peptides is limited, primarily focusing on structural investigations [25–27]. A single paper described the function of secapin-2 as being responsible for hyperalgesic and edematogenic effects without hemolytic activity, mast cell degranulation, or chemotaxis [28]. The exact mechanism by which secapin-2 induces pain and edema is not fully understood. However, in our electrophysiological TRPV1 bio-assay, it became clear that the secapin-like peptide, TPTX_1_-Tp1, in *T. processionea* venom could potentiate TRPV1 channels in the presence of capsaicin (EC_50_ = 44 ± 0.2 nM). More specifically, it reverts the desensitized TRPV1 channel to a sensitized, stabilized and active open state. In this active open state, there is a flow of monovalent and divalent cations, such as Ca^2+^ and Na^+^ into the cell. This influx results in an increase of positive ions within the cell, leading to cell depolarization. In turn, this depolarization triggers excitation in nociceptive nerve terminals, causing a sensation of itch [29]. Additionally, the influx of Ca^2+^ triggers downstream signaling events linked to the release of substance P and calcitonin gene-related peptide. Substance P and calcitonin gene-related peptide are neuropeptides that play a crucial role in the transmission of pain signals and are involved in various physiological processes such as inflammation and itch [30, 31]. This sensitized state may explain the duration of the clinical profile or the prolonged sensation of itch, pain, inflammation in vivo*.*

The fact that TPTX_1_-Tp1 lacks an impact on its own but produces an effect when combined with the agonist led us to label this phenomenon as a “foot-in-the-door effect”. This effect mirrors the observations in jellyfish venom [32]. The question now arises: how does TPTX_1_-Tp1 trigger this modulation of TRPV1? While the human body may lack capsaicin, the TRPV1 channel can still be primed (resembling an allosteric effect) through alternative means, such as the presence of anandamide (an endocannabinoid and well-studied agonist of TRPV1) or by a normal physiological body temperature of 37 °C [33]. When combined with TPTX_1_-Tp1, this priming may be efficient enough to replicate the effects observed in our experiments with capsaicin. Exploring further, it would be intriguing to evaluate the peptide TPTX1-Tp1 at core body temperature. At 37 °C, the TRPV1 channel is primed, potentially allowing the peptide to potentiate TRPV1 without the need for a low dose of capsaicin to induce an effect. Future research should delve into this interaction. Moreover, exploring the structure–activity relationship of TPTX_1_-Tp1 could shed light on the pivotal residues crucial for its activity on TRPV1. For example, elucidating whether the modulation of capsaicin-induced TRPV1 by TPTX_1_-Tp1 relies on the presence of a single pair of disulfide bridges may uncover key structural determinants governing its functionality. In a recent study by Fan Yang group it was found that the combination of capsaicin with the linear s-RhTx (a toxin from the Chinese red-headed centipede) did not potentiate TRPV1 activation whereas the folded s-RhTx in solution with capsaicin significantly potentiated the activation of TRPV1 channel to induce spontaneous pain in vivo [34]. Moreover, they found that the combined application of capsaicin and s-RhTx (a positive allosteric modulator of TRPV1) was even more effective for chronic pain relief compared with only capsaicin [34]. Since the results of our study suggest that TPTX_1_-Tp1 might be a positive allosteric modulator of TRPV1 and the fact that local application of capsaicin has been a common analgesic treatment in chronic management, TPTX_1_-Tp1 may become a new candidate drug for treating chronic pain [34].

Interestingly, our experiments have demonstrated that a TRPV1 antagonist can completely block the effects of the venom extract from *T. processionea*. The results are consistent with the discoveries presented by Yao et al. (2019) regarding *Latoia consocia*, a caterpillar indigenous to South-West China. In this context, TRPV1 played a crucial role as a nociceptor in the pain induced by the caterpillar, with a notable reduction observed when treated with capsazepine [35]. Despite substantial investments in the quest for TRPV1 antagonists, yet no antagonist is available on the market. Presently, a promising topical application, Asivatrep, is undergoing phase III clinical trials, showing encouraging results and a favorable safety profile for individuals with atopic dermatitis [36]. If the results continue to go in a positive direction, this compound has the potential to become a preferred therapeutic option for managing atopic dermatitis in the future, and perhaps also serve as a potential treatment for *T. processionea* envenomation [36].

Alternatively, an indirect interaction between TPTX_1_-Tp1 and TRPV1 can be hypothesized. TPTX_1_-Tp1 might interact with a GPCR linked to G_q_ to stimulate the phospholipase C (PLC) activity. This PLC activity cleaves phosphatidylinositol 4,5-bisphosphate (PIP_2_) into diacylglycerol (DAG) and inositol (1,4,5)-trisphosphate (IP_3_), thereby reducing the capsaicin threshold [37, 38]. This process could subsequently modulate more TRPV1 channels at the same capsaicin concentration, resulting in a heightened inward current [37, 38]. Another proposed mechanism involves the interaction of TPTX_1_-Tp1 with the EGF receptor to stimulate tyrosine kinase activity and thus the calcium-dependent phospholipase A_2_ (PLA_2_) activity [39]. Activation of the PLA_2_ pathway leads to the liberation of arachidonic acid, which, in turn, undergoes metabolism by lipoxygenases (LOX). This enzymatic process results in the formation of bioactive lipid mediators, including hydroperoxyeicosatetraenoic acid (HPETE), serving as a TRPV1 agonist [40]. If this signaling pathway is indeed functional, TPTX_1_-Tp1 indirectly triggers edema and itch sensations through TRPV1 signaling. These effects may be alleviated by Zileuton, a 5-lipoxygenase blocker, previously demonstrated to suppress pain and edema responses induced by secapin-2 injection [28]. Understanding the regulatory mechanism of PLA_2_ activity remains elusive. On the other hand, arachidonic acid can also produce prostaglandin E2 through the actions of cyclooxygenase (COX) enzymes [39]. This prostaglandin E2 can interact with GPCRs linked to G_s_, leading to an increase in intracellular cyclic AMP (cAMP) levels [39]. This cAMP activates protein kinase A (PKA), which subsequently phosphorylates TRPV1 channels [41, 42]. In the experiments with secapin-2, it was shown that indomethacin (a nonspecific antagonist of cyclooxygenase) did not show any reducing effects on edema formation and pain sensation [28]. These observations contradict the notion that the COX pathway is involved but further research is warranted to elucidate this.

Further, an increasing number of clues seem to be pointing towards a TRPV1 mechanism. In the venom of *T. processionea,* we have identified putrescine, a compound known to modulate TRPV1 channels. Putrescine is a small aliphatic polyamine synthesized by ornithine decarboxylase (ODC) and found in the venom of other animals [43]. Under physiological conditions, putrescine undergoes complete protonation [44]. This property enables putrescine to interact with negatively charged macromolecules, such as DNA, RNA, ATP, phospholipids, or proteins [44]. As a result, it plays a significant role in various biological processes, including the modulation of enzyme activities and regulating cell proliferation and differentiation [45]. In a study by Ahern et al. (2005), it was found that 5 mM putrescine alone did not induce a detectable TRPV1 activity but remarkably intensified the current evoked by 30 nM capsaicin in HEK293 cells [44]. Having the capability to regulate TRPV1 channels, putrescine emerges as a physiologically relevant modulator with the potential to induce inflammation and nociception. This was demonstrated in a different study, wherein putrescine was administered via subcutaneous injection into the skin of a rat paw [46]. A dose of 10 µmol/paw resulted in the induction of mechanical allodynia and sustained edema for a duration of 6 h [46]. Moreover, this short-chain polyamine strongly affects the biological activity of histamine by inhibiting histamine-N-methyltransferase activity [47]. It could be that when putrescine is injected into the skin, an impaired histamine metabolism takes place, which will impede the breakdown of histamine, potentially leaving more histamine in the skin than usual. This may result in an increased presence of histamine-like effects such as redness, itching, and swelling [47].

TRPV1 channels can be implicated in both the histaminergic pathway, where histamine is involved, and the non-histaminergic pathway, enabling C-fibers to convey itch without histamine involvement [48]. An intriguing facet of this non-histaminergic pathway lies in the absence of an axon reflex flare, commonly recognized as redness on the skin, akin to the initial phase of *T. processionea* envenomation [49]. Our study extensively explored the engagement of histamine and histamine receptors in the mechanism of action of *T. processionea*. Our findings reveal an absence of the small molecule histamine in the venom, and the whole venom setae extract could not activate the H1 and H4 receptors associated with itching and inflammation. This aligns perfectly with the delayed onset of symptoms, manifesting only 6–8 h after contact with the setae. Unlike typical hypersensitivity reactions elicited by histamine, such as those seen in wasp stings or mosquito bites, *T. processionea* is likely to trigger a delayed hypersensitivity reaction. However, this does not rule out the potential indirect involvement of histamine or mast cell activation in the manifestation of symptoms. Lamy et al. (1985) saw an IgE-independent effect on mast cells for thaumetopoein, the first component found in *T. pityocampa*. Despite this, thaumetopoein was never sequenced so no further information is available [50]. All of this might explain the reason behind the ineffective relief of *T. processionea* symptoms by current antihistaminic drugs.

However, future research is imperative to fully elucidate how TPTX_1_-Tp1 and the other venom components of *T. processionea* contribute to pain, itch, and allergic reactions associated with lepidopterism. For instance, animal studies could provide insights into the clinical effects of TPTX_1_-Tp1. By observing the physiological responses in vivo, such as changes in behavior, sensory perception, and inflammatory markers, we can gain a better understanding of the overall impact of TPTX_1_-Tp1 on the organism. Moreover, it is also essential to investigate the effect of the crude venom extract from *T. processionea* or TPTX_1_-Tp1 on peripheral neurons and mast cells. On these peripheral neurons and mast cells, several itch receptors (TRPV1, TRPA1, Na_v_1.7, H1R, MRGPRs, and PARs) are responsible for the sensation of itch [51–54]. Following the methodologies outlined in Robinson et al. (2018), in the future, researchers may perform calcium imaging experiments using sensory neurons from the mouse dorsal root ganglion (DRG) to elucidate cellular depolarizations or whole cell patch-clamp electrophysiology to further assess the impact of TPTX_1_-Tp1/other venom components on these itch receptors [55]. Additionally, it's essential to assess whether TPTX_1_-Tp1 or other venom components have the potential to directly interact with mast cells through MRGPRs or PARs and stimulate mast cell degranulation. Mast cell degranulation releases substances that triggers allergic reactions.

## Conclusion

In summary, our research emphasizes the pivotal role of TPTX_1_-Tp1 within the urticating setae of *T. processionea* in modulating TRPV1 receptor activity. The predicted 3D structure of TPTX_1_-Tp1, resembling secapin peptides found in bee venom, highlights an evolutionary adaptation where peptides involved in normal insect physiology serve as modulators of itch, inflammation, and pain. Although TPTX_1_-Tp1 demonstrates unique effects on TRPV1 modulation, uncovering the specific binding site or interaction mechanism remains a challenge for future research. The widespread presence of caterpillars with urticating setae and the frequent occurrence of skin irritation drive the demand for the development of an innovative topical medication. This study lays the foundation for the creation of topical medication, proposing the inclusion of a TRPV1 blocker to address the localized effects induced by *T. processionea*. The hope is that ongoing research on Asivatrep or other topical TRPV1 blockers advances not only in addressing atopic dermatitis but also in proving effective against the venomous effects of caterpillars. Such progress holds the promise of bringing relief to individuals in Europe and globally who encounter these caterpillars during outdoor activities.

### Supplementary Information

Below is the link to the electronic supplementary material.

Supplementary file1 (XLSX 197 KB)

Supplementary file2 (DOCX 2684 KB)

## Data Availability

The datasets generated during and/or analyzed during the current study are available from the corresponding author on reasonable request.
