# Supplementary material for: Exploring oak processionary caterpillar induced lepidopterism (part 2): ex vivo bio-assays unmask the role of TRPV1
Source: Cell Mol Life Sci. 2024 Jun 28;81(1):281. doi: 10.1007/s00018-024-05318-9 (PMC11335206; doi:10.1007/s00018-024-05318-9)
Supplement: Supplementary file 2 — Supplementary file2 (DOCX 2684 KB) [file 18_2024_5318_MOESM2_ESM.docx]

Exploring Oak Processionary Caterpillar Induced Lepidopterism (Part2): *Ex vivo* Bio-assays Unmask the Role of TRPV1

**Andrea Seldeslachts**^1^, Eivind Andreas Baste Undheim^2^, Joris Vriens^3^, Jan Tytgat^1*^, Steve Peigneur^1,3*^

*^1^Laboratory of Toxicology and Pharmacology, Department Pharmaceutical and Pharmacological Sciences, Catholic University of Leuven, Leuven, Vlaams-Brabant, Belgium*

*^2^Centre for Ecological and Evolutionary Synthesis, Department of Biosciences, The University of Oslo, Oslo, Norway*

*^3^Laboratory of Endometrium, Endometriosis and Reproductive Medicine, Department of Development and Regeneration, KU Leuven, Leuven, Belgium.*

*Correspondence: [steve.peigneur@kuleuven.be](mailto:steve.peigneur@kuleuven.be); [jan.tytgat@kuleuven.be](mailto:jan.tytgat@kuleuven.be);

Supplementary Material

# Supplementary Figures


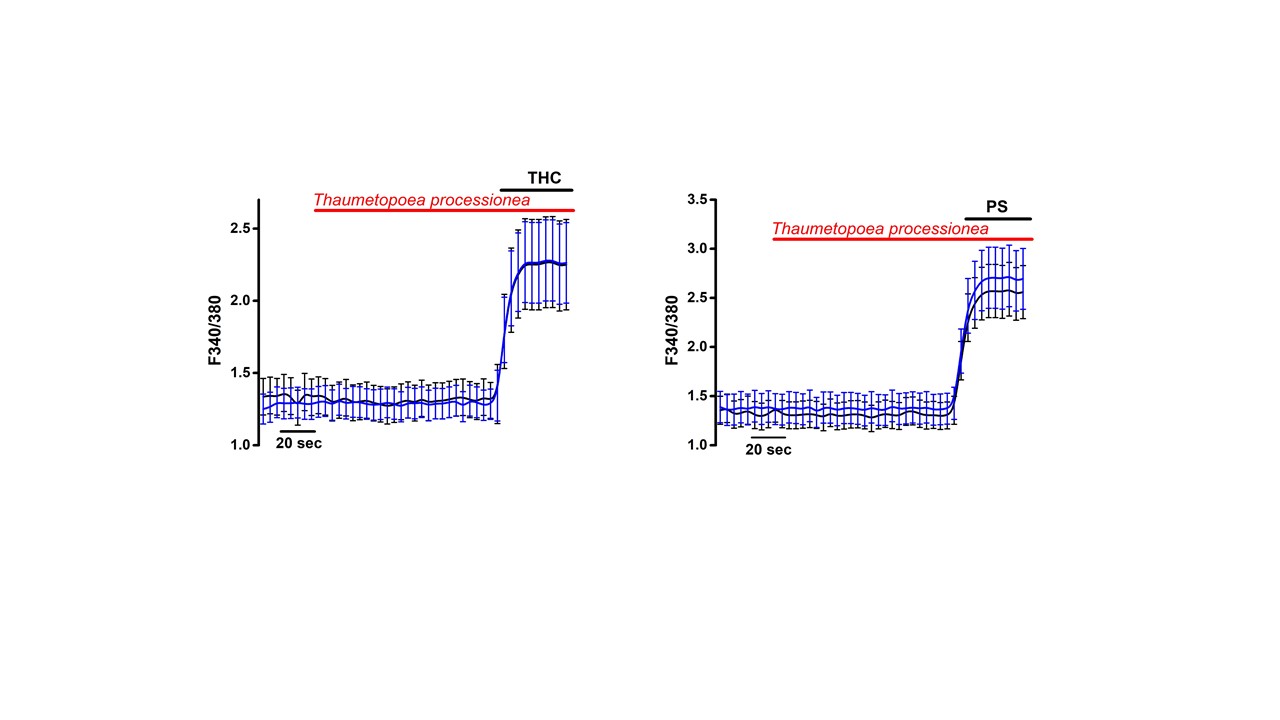


***Supplementary Fig. S1 Effect by crude Thaumetopoea processionea venom setae extract on mTRPV2 (left panel) and hTRPM3 (right panel)*** ***in human embryonic kidney cells (HEK) cells.*** *Ratio traces (F340/380) of T. processionea venom setae extract (final concentration of 0.5 µg/µL) and vehicle as observed in the Flexstation. Venom or vehicle were applied after 20 seconds and THC (50 µM, positive control) or PS (40 µM, positive control) were applied at 140 seconds. The black line indicates vehicle, the blue line indicates venom.*


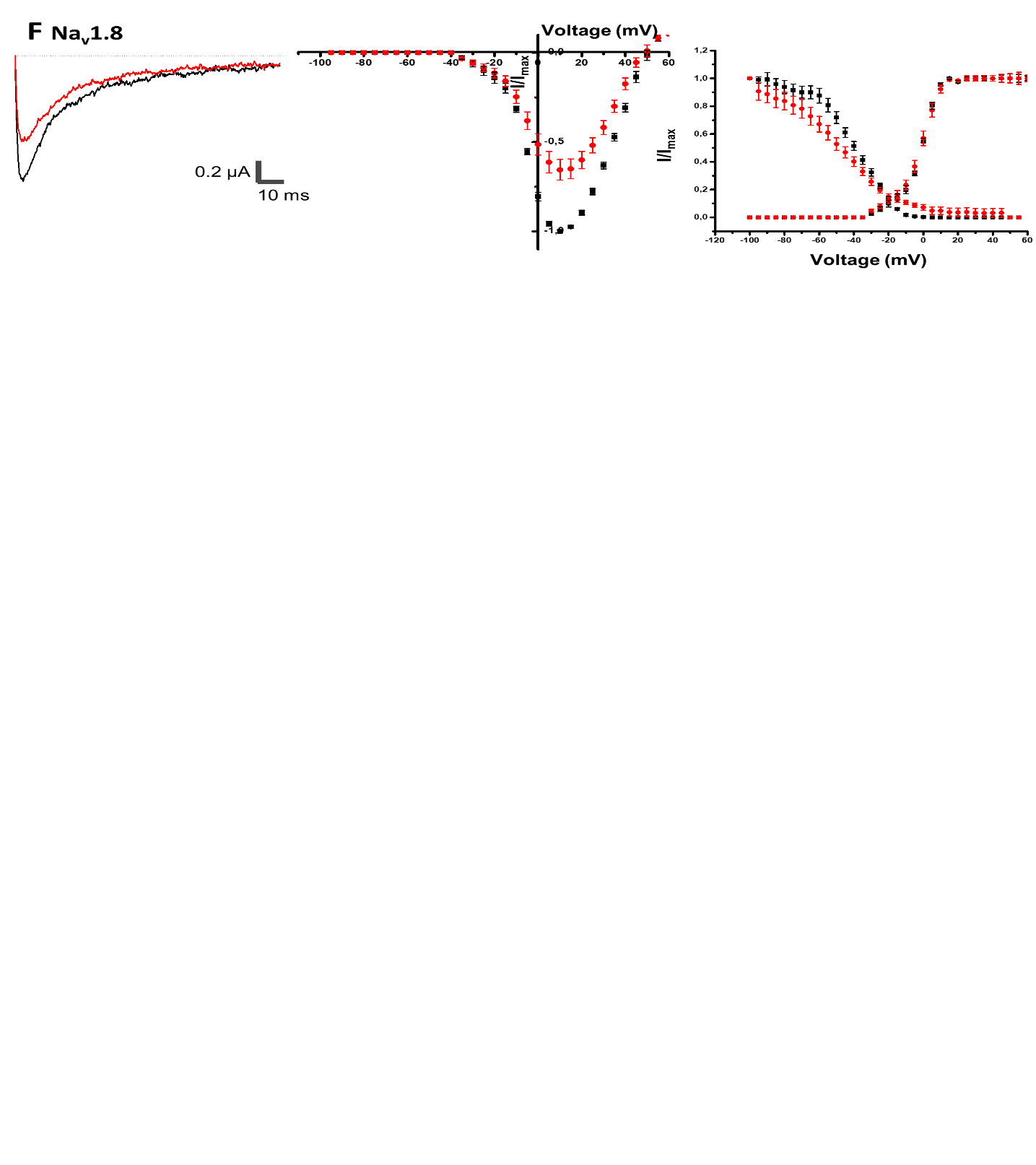

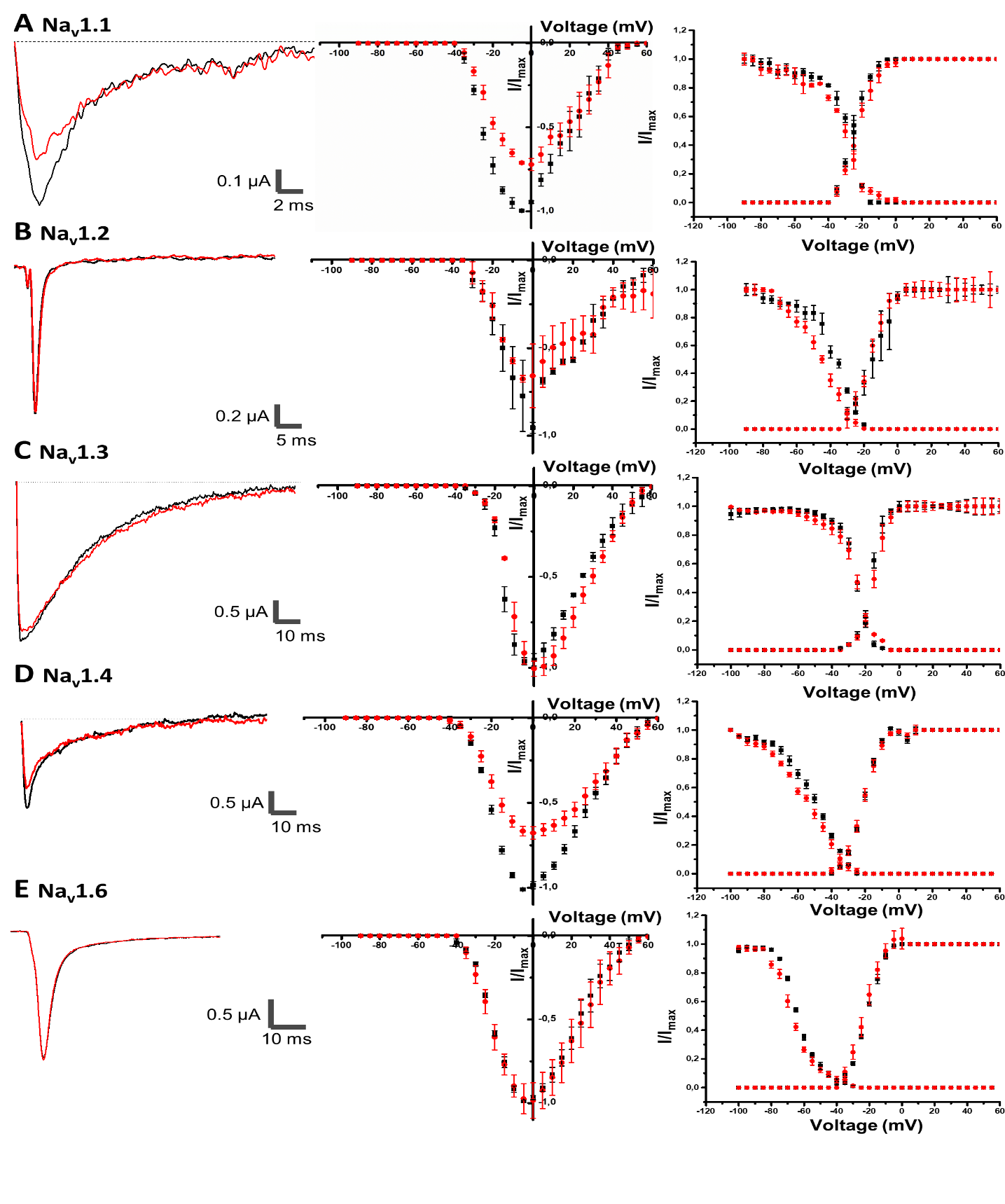


**0.4 µg/µL TP**

**ND96**

*Supplementary Fig. S2 Electrophysiological characterization of Thaumetopoea processionea venom extract on mammalian Na_v_ channels. The left and the middle panels show the current voltage relationship and the right panel represents the steady-state activation and inactivation curves in control ND96 buffer (black) and toxin condition 0.4 µg/µL T. processionea venom extract (red) for (A) Na_v_1.1, (B) Na_v_1.2, (C) Na_v_1.3, (D) Na_v_1.4, (E) Na_v_1.6, (F) Na_v_1.8.*


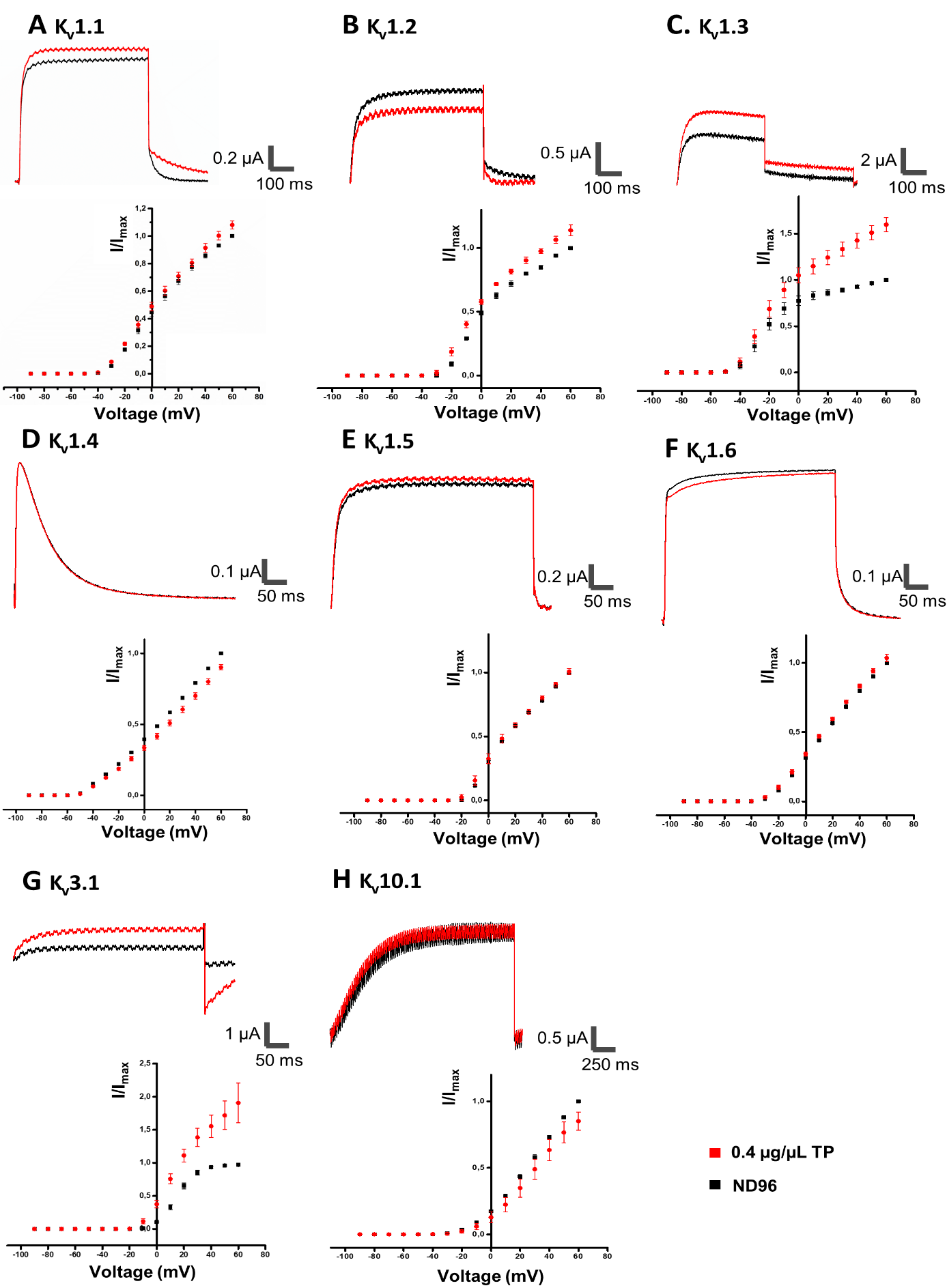


*Supplementary Fig. S3 Electrophysiological characterization of Thaumetopoea processionea venom extract on K_v_ channels. The black line represents the control ND96 buffer condition and the red line indicates the currents obtained in the presence of 0.4 µg/µL T. processionea venom extract (red) for (A) K_v_1.1, (B) K_v_1.2, (C) K_v_1.3, (D) K_v_1.4, (E) K_v_1.5, (F) K_v_1.6, (G) K_v_3.1, (H) K_v_10.1.*


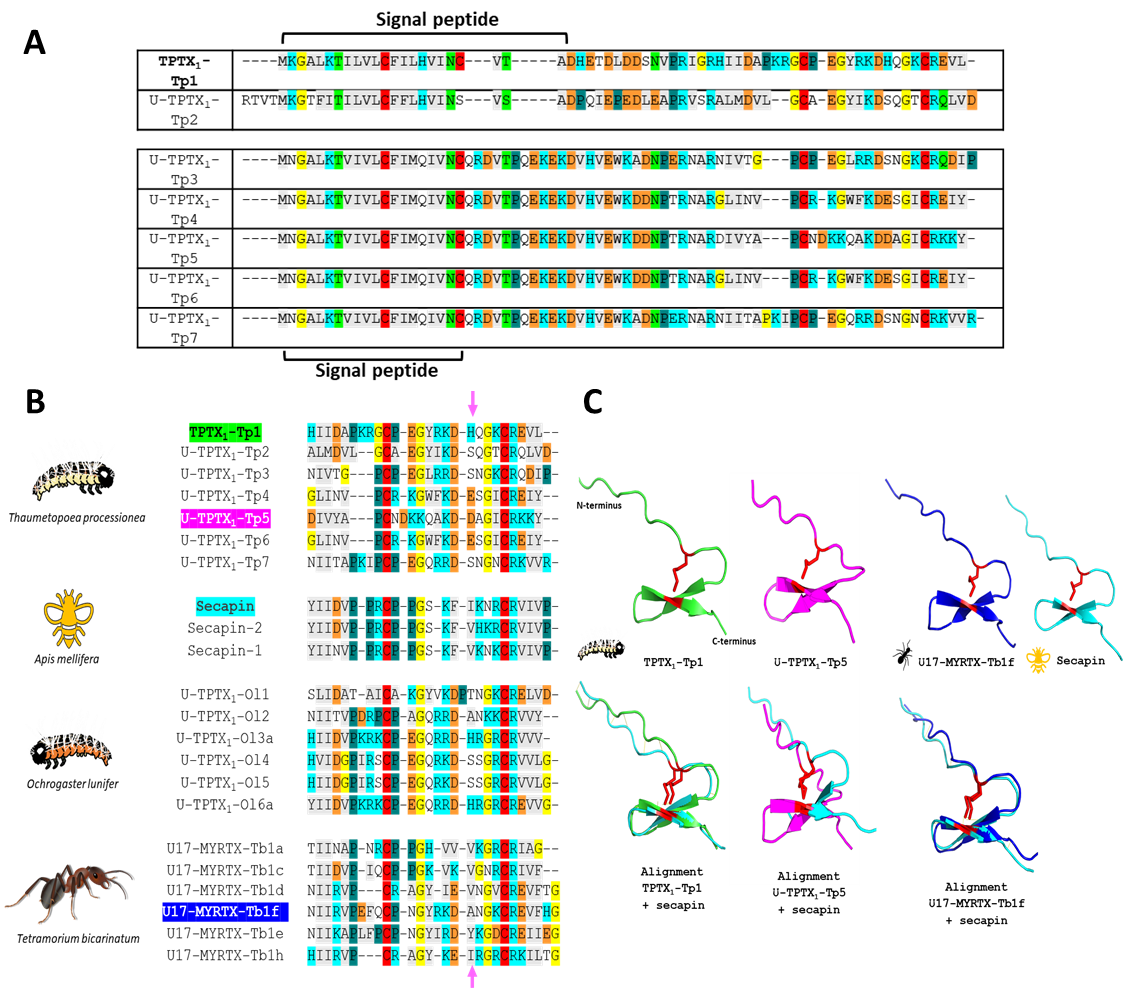


*Supplementary Fig. S4 Secapin-like peptide family in the venom of Thaumetopoea processionea. (A) Alignment of T. processionea secapin-like peptides with signal P-sequence. (B) Alignment of T. processsionea peptides with secapins from Apis mellifera, secapin-like peptides from Ochrogaster lunifer and Tetramorium bicarinatum. (C) AlphaFold2 predicted 3D structure of TPTX1-Tp1 and U-TPTX1-Tp5 from T. processionea compared with a secapin-like peptide U17-MYRTX-Tb1f from Tetramorium bicarinatum and compared and aligned with secapin from Apis mellifera.*

# Supplementary information

#### Secapin-like peptides

We identified seven secapin-like peptides ((U)-TPTX_1_-Tp(1-7)) that all share a common cysteine scaffold (**Fig. 2A**). Confirmation of their presence in the venom of *T. processionea* was achieved through the proteotranscriptomic pipeline, revealing high -10lgP scores. For example, TPTX_1_-Tp1 exhibited a -10lgP score of 260.68, and U-TPTX_1_-Tp5 showed a -10lgP score of 214.36. A Blastp search revealed that TPTX_1_-Tp1 shares 81.2% similarity with U17 myrmicitoxins, which are identified in ant venom and classified as secapin-like peptides. Aligning (U)-TPTX_1_-Tp(1-7) found in *T. processionea* venom with secapins from *Apis mellifera*, secapin-like peptides from *Ochrogaster lunifer,* and myrmicitoxins from *Tetramorium bicarinatum* revealed shared features suggesting a common ancestor. These include conserved residues and a highly similar structural fold characterized by a single disulfide bridge that likely plays a crucial role in sustaining a high degree of structural stability (**Fig. 2B**). In this context, a pair of amino acids with hydrophobic side chains resemble the N-terminus (grey), and an arginine-lysine-rich (light blue) region is visible around the second cysteine. Around the first cysteine, almost all sequences have a proline (green) and a conserved glycine (yellow) residue. From the overall domain architecture, we can tell that most molecules are highly charged. For example, TPTX_1_-Tp1 has 3 arginine residues and 2 lysine residues, while U-TPTX_1_-Tp5 has 1 arginine residue and 5 lysine residues. Moreover, we want to highlight the residues indicated with a pink arrow on the top and bottom of the alignment. For TPTX_1_-Tp1, a positive charge, and for U-TPTX_1_-Tp5, a negative charge was visible, while secapin and U17-MYRTX-Tb1f both have a hydrophobic residue that has previously been found to be under positive selection [1]. According to Barassé *et al.* (2022), a variation in charge and polarity on this specific position could potentially influence the conformation and functionality of the peptides [1]. In addition, (U)-TPTX_1_-Tp(1-7) lack the amidation tag, characterized by the absence of the glycine (G) or the basic residues lysine-arginine (KR) or lysine (K). These findings suggest that the peptides don’t possess the necessary features for C-terminal amidation in contrast with the peptides found in the venom of *O. lunifer* and *T. bicarinatum* [1,2].

To further compare the structural similarities of venom secapins, the 3D structures of TPTX_1_-Tp1 and U-TPTX_1_-Tp5 were predicted using AlphaFold2 (**Fig. 2C**). In these 3D structures, the key common structural feature is a double-stranded antiparallel β-sheet connected by a β-turn, also known as a β-hairpin motif. Both the N-terminus and C-terminus exhibit an unstructured tail. The same 3D structure was also described for U17-MYRTX-Tb1a and U-TPTX_1_-Ol1 [1,2]. Furthermore, the RMSD value of 2.342 Å, calculated for 137 aligned atoms, indicates a relatively high level of structural similarity between the predicted mature molecular structures of TPTX_1_-Tp1 from *T. processionea* and secapin from *A. mellifera*. For U-TPTX_1_-Tp5 from *T. processionea* and secapin from *A. mellifera*, a close match between the atoms in the mature structures was obtained (RMSD of 0.136 Å). These findings point to a remarkable degree of similarity and consistency between (U)-TPTX_1_-Tp(1-7), U17-MYRTX-Tb, U-TPTX_1_-Ol, and secapin.

1. Barassé V, Téné N, Klopp C, Paquet F, Tysklind N, Troispoux V, et al. Venomics Survey of Six Myrmicine Ants Provides Insights into the Molecular and Structural Diversity of their Peptide Toxins. *Insect Biochem Mol Biol* 2022, *151*, 103876. , DOI: 10.1016/j.ibmb.2022.103876.

2. Walker AA, Perkins LE, Battisti A, Zalucki MP, King GF. Proteome of Urticating Setae of Ochrogaster lunifer, a Processionary Caterpillar of Medical and Veterinary Importance, including Primary Structures of Putative Toxins. *Proteomics* 2023, *23*, e2300204. , DOI: 10.1002/pmic.202300204.
